# Establishing an EU-compliant diagnostic facility for infectious diseases under war conditions in Poltava, Ukraine

**DOI:** 10.3389/fpubh.2026.1840777

**Published:** 2026-05-29

**Authors:** Gennadiy Zelinskyy, Tetiana Koval, Ursula Schrammel, Carina Elsner, Jessica Ackermann, Olena Marchenko, Mykhailo Sydorenko, Oksana Shlykova, Olga Izmailova, Alina Kabaliei, Jan Dziobaka, Hedda Luise Verhasselt, Jan Buer, Evelyn Heintschel von Heinegg, Mirko Trilling, Igor Kaydashev, Ulf Dittmer

**Affiliations:** 1 Institute for Virology, University Hospital Essen, University of Duisburg-Essen, Essen, Germany; 2 Department of Infectious Diseases with Epidemiology, Poltava State Medical University, Poltava, Ukraine; 3 Poltava Regional Clinical Infectious Diseases Hospital of the Poltava Regional Council, Poltava, Ukraine; 4 Research Institute of Genetic and Immunological Bases of Pathology and Pharmacogenetics, Poltava State Medical University, Poltava, Ukraine; 5 Institute of Medical Microbiology, University Hospital Essen, University of Duisburg-Essen, Essen, Germany; 6 Institute for the Research on HIV and AIDS-associated Diseases, University Hospital Essen, Essen, Germany; 7 Department of Internal Medicine №3 with Physiology, Poltava State Medical University, Poltava, Ukraine

**Keywords:** diagnostic, HBV, HCV, HIV, infectious diseases, antimicrobial resistance (AMR), technology transfer

## Abstract

Russia’s invasion has systematically destroyed Ukrainian healthcare infrastructure while simultaneously increasing infectious disease risks through wounded combatants and civilians, people displacement, and disrupted care. Poltava, a central region hosting over 200,000 internally displaced persons, already faced pre-war infectious disease incidences higher than the national average, yet diagnostics relied mostly on low-sensitivity rapid tests. Through a German-Ukrainian hospital partnership funded by the Deutsche Gesellschaft für Internationale Zusammenarbeit, we established EU-compliant infectious disease serology (ELFA) and automated PCR, as well as an automated bacterial identification system with antibiotic susceptibility testing in Poltava’s Regional Clinical Infectious Diseases Hospital. Key elements of the partnership included participatory equipment selection, intensive hands-on training of Ukrainian staff in Germany, joint standard operating procedures, and adaptive reagent supply. Between March 2024 and June 2025, the laboratories in Poltava performed 16,633 tests, detecting previously unrecognized HIV, Hepatitis B and C, Lyme disease, Toxoplasmosis, and antibiotic-resistant bacterial infections. This project demonstrates that advanced diagnostic capacities can be established under war conditions when partnership design prioritizes easy and strait forward solutions, shared decision-making, and contingency planning. The prototypical establishment of powerful serological and molecular diagnostics in infectious diseases through this German-Ukrainian partnership may serve as a model for the implementation in other regions of the Ukraine. Even under the difficult conditions of war, this advancement in infectious disease diagnostics allows for more targeted patient care and contributes to the prevention of pathogen transmission in the Ukraine.

## Introduction

To cope with the Russian aggression, the Ukraine shifted a relevant fraction of their available medical capacities to the immediate care for people being affected by military operations, such as wounded combatants and civilians, in addition to standard care of patients (1). This is obviously a crucial and necessary immediate reaction in a war scenario. Additional problems for the Ukraine healthcare system are the destruction of hospitals and medical facilities (occurring at least in relevant parts deliberately) (2, 3) and the shortage of medical staff, because highly qualified medical personnel left the country or joined the military. This makes medical care in Ukraine increasingly difficult. In addition, it diverts attention from important medical issues such as infectious diseases, which often accompany traumatic injuries (4, 5). These problems are further exacerbated by the destruction of civil infrastructure. In many previous military conflicts it has been shown that infectious diseases are the second most relevant health threat besides traumata (4, 6). We know that there is a substantial threat that infectious disease control may be severely compromised in the Ukraine during the ongoing war (7).

In many hospitals and laboratories in the Ukraine, the diagnosis of infectious diseases and the proper management of chronic communicable infections, excluding tuberculosis (TB) and human immunodeficiency virus (HIV), are currently limited due to a lack of suitable laboratory testing equipment, as well as insufficient experience with state-of-the-art diagnostic tests. For example, TB and HIV diagnostics were provided by USAID-funded programs only at specialized HIV and TB centers (8). However, broad molecular diagnostic is an indispensable first step in treating infectious diseases. Only after identification of the disease-causing pathogen, adequate and effective treatment can be initiated. This should be an important goal for all hospitals in the Ukraine and not only for special centers. Powerful diagnostics are especially relevant for people who have been wounded in military action because these patients often suffer from pathogen-contaminated wounds (9, 10) and have an overall increased risk of acquiring infectious diseases (9). In the context of war, increasing numbers of bloodborne infections are expected (10, 11, 12). Prior to the Russian invasion, the Ukraine already recorded some of the highest prevalence rates in Europe for key bloodborne viruses, such as HIV, Hepatitis B (HBV), and Hepatitis C (HCV) (13). The epidemiological situation became even more challenging during the war because population displacement, interruption of healthcare services, and deterioration of living conditions facilitated the transmission of blood-borne and opportunistic infections.

This project was performed in Poltava, Ukraine. Poltava is the largest regional center without direct contact with the front line. It is geographically surrounded by the Sumy, Kharkiv, and Dnipro regions—all experiencing active hostilities—making it the first major safe city for civilians fleeing conflict and combatants rotating away from the front. This convergence has resulted in over 200,500 internally displaced persons and thousands of military personnel residing in the Poltava region (14). They often live in suboptimal housing conditions characterized by overcrowding, shared spaces, and limited excess to personal hygiene items. These conditions facilitate parenteral and mucosal exposure to blood-borne viruses. Consequently, Poltava serves as a critical convergence point where conflict-driven displacement, substandard living conditions, and disrupted access to infection prevention and treatment create an elevated, multi-directional transmission network for HIV, HBV, and HCV. This convergence created increased pressure on the regional healthcare system and altered local epidemiological conditions, particularly regarding blood-borne viral infections and antimicrobial resistance.

In the years before the COVID-19 pandemic, HBV and HCV infection rates remained relatively stable, but reported infections declined markedly during the pandemic due to reduced social contact and limited access to testing during lockdowns (15). However, as the pandemic eased and the war began, the incidence of both HBV and HCV began to rise again, reflecting the combined effects of population displacement, disruptions in the healthcare system, and increased transmission risks (15).

In 2017, the incidence of HBV in Ukraine (3.6 per 100,000) was lower than the EU/EEA average of 6.7 per 100,000 (16). However, the Poltava region reported a notably higher rate of 7.7 per 100,000, surpassing the EU/EEA average (17). By 2023, while the EU/EEA rate increased to 8.1 per 100,000 (18) and Ukraine’s national rate remained stable at 3.4 per 100,000, but the Poltava region continued to exhibit an elevated incidence of 7.2 per 100,000 (17). This persistent disparity indicates a distinct epidemiological pattern in Poltava compared to both national and European trends. This disparity is even more pronounced for HCV. In 2017, the EU/EEA rate was 7.3 per 100,000 (19), while Ukraine’s rate was already higher at 13.7 per 100,000. By 2023, the EU/EEA rate remained stable at 7.4 per 100,000 (20), but Ukraine’s incidence increased to 14.9 per 100,000. Strikingly, the Poltava region experienced a dramatic surge from 24.5 per 100,000 in 2017 to 45 per 100,000 in 2023 (15). This rate is more than six times the EU/EEA average and underscores the critical public health challenge in the region. For HIV in 2023, Ukraine reported a rate of 31.7 per 100,000 (21), which is among the highest in the WHO European Region. In stark contrast, the EU/EEA average rate was significantly lower at 5.3 per 100,000. This nearly six-fold difference highlights the disproportionate burden of HIV in Ukraine relative to Western European countries. In the Poltava region, the incidence of HIV increased from 26.1 per 100,000 population in 2017 to 45.7 per 100,000 in 2023, reflecting a marked upward trend that contrasts with the overall situation in the EU/EEA, where the average HIV diagnosis rate in 2023 was 5.3 per 100,000 population. Within the EU, the lowest national rates were below 1 per 100,000 population, while the highest were above 9.9 cases reported in Latvia (21).

The numbers make it clear that the rates of HBV, HCV, and HIV have always been higher in the Poltava region than in Ukraine as a whole. Therefore, the data show how the dynamics of blood-borne infections in Ukraine changed during the pandemic and the war that followed. These epidemiological developments underline the urgent need for strengthened regional infectious disease diagnostics and antimicrobial resistance surveillance capacities in central Ukraine.

### Context

Besides combatants, women and children are endangered by infectious diseases, as they represent the biggest group of internally displaced people from the eastern parts of the Ukraine, where the general healthcare system has broken down. Stress, malnutrition, destruction of civilian infrastructure, disruption of local transportation and communication, destruction of medical facilities, and lack of medical assistance in territories temporarily not under the control of the Ukraine Government are the most serious problems affecting patients in the Ukraine (6, 22). Such individuals have an increased risk to become seriously infected and will profit from the improvement of and access to state-of-the-art routine diagnostic services. This also applies to geriatric care facilities, facilities for handicapped people and orphanages.

The awareness for diagnostics of communicable diseases as well as the prevention of the spread of pathogens such as HIV, HBV, HCV, and multi-resistant bacteria within the Ukraine is also highly important for all European countries welcoming refugees from the Ukraine. As example, in March 2025 more than 6.37 million refugees have been recorded in Europe and more than half a million individuals beyond Europe (23). Based on UNAIDS data indicating that there were 244,900 (approximately 0.6% of the population) people living with HIV (PLWH) in 2021 in the Ukraine, the European Centre for Disease Prevention and Control (ECDC) estimated that between 9,000 and 56,000 PLWH came to Europe (24, 25). From these refugees living with HIV many are undiagnosed or live with untreated or insufficiently treated HIV (26). Accordingly, there was a steep increase in the number of initial HIV diagnoses and detection of drug resistant HIV (27) in Germany associated with a refugee background from Ukraine. Furthermore, highly virulent and pan-resistant strains of *Klebsiella pneumoniae* of Ukrainian origin have been observed in several European countries (28–31). Ukraine faces a critical convergence of two threats: a severe shortage of last-resort antibiotics such as Carbapenems due to war-related supply disruptions, and one of the highest rates of antimicrobial resistance (AMR) in Europe. Pre-war data from 2020 already demonstrated alarming resistance levels. A study on war-injured patients from Eastern Ukraine confirmed a broad spectrum of resistance-associated genes in *Acinetobacter baumannii* (32), and subsequent surveillance reported by the Center for Public Health of Ukraine found that up to 74.7% of *A. baumannii* isolates were resistant to Carbapenems and 77.5% of *Klebsiella pneumoniae* isolates were resistant to third-generation Cephalosporins (33). More recent data from the Ukrainian front line reveals that Carbapenem resistance remains highly prevalent, and isolates of *K. pneumoniae* from war victims have been found to be both Carbapenem-resistant and hypervirulent, posing a significant risk (34). Furthermore, a 2025 case report documented a single severely injured Ukrainian soldier carrying 14 distinct Carbapenemase-producing microorganisms (35), highlighting the risk of rapid regional spread of these resistant organisms. Rapid pathogen identification and antimicrobial susceptibility testing (AST) that provide minimum inhibitory concentration (MIC) results within hours instead of days allow for the direct stewardship of scarce antibiotic resources, improving individual patient outcomes and slowing the spread of Carbapenem-resistant organisms across the region.

Diagnosing and preventing infectious diseases in the Ukraine may also impact the healthcare system in other European countries. The local war in the Donbass region in 2014–15 resulted in an HIV outbreak in other parts of the Ukraine, most likely due to internal replacement of citizens (36). Thus, bloodborne viruses have a high potential for long-distance transmission and spread to other regions or even countries during war time and the central Poltava region seems to be especially affected.

After Russia’s attack on the Ukraine, it became immediately evident that the Ukrainian healthcare system urgently needs support in order to maintain medical care in areas of routine diagnostics, medical training, and translational research as part of emergency aid measures. The University Medicine in Essen (UME), Germany, already collaborated with the Poltava State Medical University (PSMU) and decided to apply for a Hospital Partnership Programme from the *Deutsche Gesellschaft für Internationale Zusammenarbeit* (GIZ) GmbH. Because of the war, the diagnosis and treatment of infectious diseases in the Ukraine was reduced to a minimum in order to reallocate capacities for the treatment of traumata. However, infections constitute a highly relevant medical issue that needs to be taken seriously. Therefore, we initiated a hospital partnership project between UME, PSMU, and the Poltava Regional Clinical Infectious Diseases Hospital of the Poltava Regional Council in April 2023. The overall goal was to set up a state of the art, EU-compatible diagnostic center for the most common and medically relevant infectious diseases in Poltava to serve as a diagnostic hub for the whole region of central Ukraine and to optimize therapy and infection control. In addition, our efforts aimed at preparing the Poltava laboratories for requirements and standards for EU accession of the Ukraine. Strengthening reliable, EU-compliant laboratory diagnostics in this region is therefore essential to maintain continuity of care for both resident communities and displaced populations during the ongoing war (37). It is crucial to emphasize the alignment of diagnostic methods and infection control protocols with the most recent EU clinical guidelines and standardized performance criteria, ensuring exceptional sensitivity, specificity, and reliability in pathogen detection. Focus on point-of-care testing technologies that empower timely diagnosis and effective infection containment, especially vital in regions near conflict zones or those with disrupted healthcare systems. The integration of infection diagnostics in Poltava at both the Ukrainian national and EU levels is essential to enable real-time tracking of infectious disease outbreaks, providing data for epidemiological studies and enabling swift public health responses. Last but not least, we wanted to provide diagnostics for infectious diseases in all relevant patient groups, as the diagnosis and treatment of infections should be independent of the economic situation of the patient.

The partner institutions in the Poltava region were the Poltava State Medical University (PSMU) and the Poltava Regional Clinical Infectious Diseases Hospital. Founded in 1921, PSMU is one of Ukraine’s oldest medical universities, training over 5,000 students across various health disciplines and conducting government-funded research, clinical trials, and large-scale biomedical studies. The Poltava Regional Clinical Infectious Diseases Hospital serves as PSMU’s clinical base, specializing in treating infectious diseases—including Hepatitis, HIV, and COVID-19—with 152 beds and a multidisciplinary medical team. During the pandemic, the hospital had to focus on COVID-19 care, reducing overall patient numbers, but by 2024 it had resumed broader infectious disease services and treated nearly 4,000 patients, although modernization of its diagnostic units were urgently needed.

## Aims

The project aimed to establish sustainable, EU-compatible diagnostic capacities for clinically relevant infectious diseases under wartime conditions in central Ukraine. The specific objectives were:

- To establish and implement modern serological, molecular, and bacteriological diagnostic infrastructure for infectious diseases in the Poltava region.
- To strengthen sustainable local laboratory capacities through technology transfer, staff training, and implementation of standardized operating procedures aligned with EU-oriented laboratory quality principles.
- To improve rapid pathogen detection and antimicrobial resistance diagnostics in order to optimize patient management and infection control under wartime conditions.
- To support regional public health preparedness and surveillance capacities for blood-borne viral infections and multidrug-resistant bacterial pathogens.
- To establish a long-term educational and scientific basis for infectious disease diagnostics, research, and medical training at PSMU.

The overall goal was to create a resilient diagnostic hub capable of maintaining high-quality infectious disease diagnostics despite war-associated disruptions of healthcare infrastructure, logistics, and routine medical services.

### Project strategy and obstacles

To achieve the overall goal of establishing a state-of-the-art, EU-compatible diagnostic center for infectious diseases in Poltava that can serve as a diagnostic hub for the entire region of central Ukraine.

Based on the lists of important pathogens and the corresponding number of diagnostic tests received from Poltava for both viral and bacterial agents, we proposed the use of an Enzyme Linked Fluorescent Assay (ELFA) technology as an initial screening approach for serological pathogen detection (*MINI VIDAS* system, bioMérieux). For confirmation of screening results and important information on pathogen loads, we selected real-time reverse transcription polymerase chain reaction (RT-PCR) (*altoStar®* Automation System, altona Diagnostics), employing an automated systems for nucleic acid isolation and the preparation of amplification mixes to ensure analytical precision and reproducibility. In the field of microbiology diagnostics, we recommended an automated system that enables both comprehensive identification of bacterial pathogens and the determination of antibiotic resistance profiles (*VITEC 2*, bioMérieux), thereby supporting evidence-based clinical and epidemiological decision-making.

We implemented of the serological and PCR diagnostic methods in our project according to the company’s regulatory standards. All test systems implemented are EC certified. The use of EC-certified assays ensures conformity with European regulatory standards regarding quality, safety, and diagnostic performance. This is important for EU integration, as it enables harmonization with established European diagnostic frameworks, facilitates interoperability and mutual recognition across member states, and supports the reliable exchange and comparability of diagnostic data within the EU public health infrastructure. The tests procedures adhere to established quality management systems and operational protocols required for compliance with EU standards. This includes the use of validated assay kits, routine inclusion of positive and negative controls in each diagnostic run, calibration with international standards, and ongoing quality assurance measures, all ensuring reliable and reproducible results as per the manufacturer’s regulations and certifications.

We defined and implemented the following steps for our Hospital Partnership Programme:

- We carefully selected diagnostic devices that could be transported, implemented, installed and operated in Poltava. Easy handling and established supply chains, as well as technical support, were important for the sustainable use of the equipment in the future.
- Intensive theoretical and practical training for colleagues from the PSMU and the KP Poltava Regional Clinical Infectious Diseases Hospital (doctors, medical staff, and employees of routine diagnostics and research laboratories) at the UME in the Institutes for Virology and Medical Microbiology. All selected diagnostic devices were available in Essen for the training.
- Acquisition of laboratory equipment for Poltava as well as the requirements in terms of reagents and consumables.
- Shipment of equipment and consumables to the two partner sites in Poltava.
- Further video training in Poltava after they received the equipment.
- Establishment of standard operation procedures (SOPs).
- Collecting reports on the diagnostic procedures performed in Poltava. Clinical trial number: not applicable.

The analysis of patients data was conducted in accordance with the ethical principles outlined in the Declaration of Helsinki (38) and received approval from the Ethics Committee of Poltava State Medical University.

Patient data were obtained from clinical diagnostic tests conducted to diagnose infections. In line with clinical practice in Poltava, adult female and male patients with suspected infectious diseases, including those who returned from frontline combat, were admitted or referred to the infectious disease hospital for diagnosis. Patient data were fully anonymized and presented in an aggregated form. The results from the diagnostic tests described in this publication were generated between March 2024 and June 2025.

The project planning was developed in close dialogue with the Ukrainian partners, and the equipment was selected according to the requirements for the ease of handling, cost-effectiveness, and sustainability. Particular attention was devoted to ensuring that all proposed solutions align with local technical capacities, maintenance frameworks, and long-term operational feasibility. The participatory approach enabled continuous adaptation of project components to the specific institutional and regional context, thereby fostering ownership, accountability, and the durability of results.

However, the implementation phase encountered several challenges. In Germany, the administrative processes within university clinics proved highly complex, with limited personnel resources and competing institutional priorities causing delays in decision-making and procurement. Simultaneously, the unexpected escalation of war-related circumstances in the Ukraine created serious logistical constraints, disrupted local supply chains, and required frequent modifications of the project timeline. The continuous uncertainty and fluctuating security conditions demanded a flexible management approach and constant coordination between partners to sustain progress under highly volatile conditions.

Prior to the hardware shipment, the staff from Poltava received an intensive, hands-on training in Essen (at the Institutes for Virology and Medical Microbiology) using the exact same devices thereafter shipped to Poltava. The successful training of Ukrainian laboratory personnel and physicians in German diagnostic laboratories was the first requirement for the implementation of the project. For laboratory personnel and physicians from the Ukraine, low English or German proficiency posed a significant obstacle to training Ukrainian personnel in Germany. To address this issue, it was arranged that there was always at least one Ukrainian participant who spoke fluently English in each small group, and these groups were further supported by German colleagues with knowledge in Ukrainian. This approach enabled effective communication and efficient training for Ukrainian staff with limited foreign language skills. Three senior physicians from the Poltava Infectious Diseases Hospital were trained for 1 week at the West German Center for Infectious Diseases [Westdeutsches Zentrum für Infektiologie (WZI)]. In total, six colleagues from Poltava, including doctors and laboratory staff, were trained for 3 weeks. The trained personnel acquired new expertise in methods, quality management and standard operating procedures (SOPs) through knowledge transfer, and were able to implement these measures in Poltava. An additional organisational challenge faced by Ukrainian male participants in the project was related to national regulations requiring men to obtain military service clearance before being granted permission to travel abroad for training. This meant that male participants had to secure official authorisation to leave the country, which introduced considerable time and administrative complexities.

### Implementation

**Figure 1:**
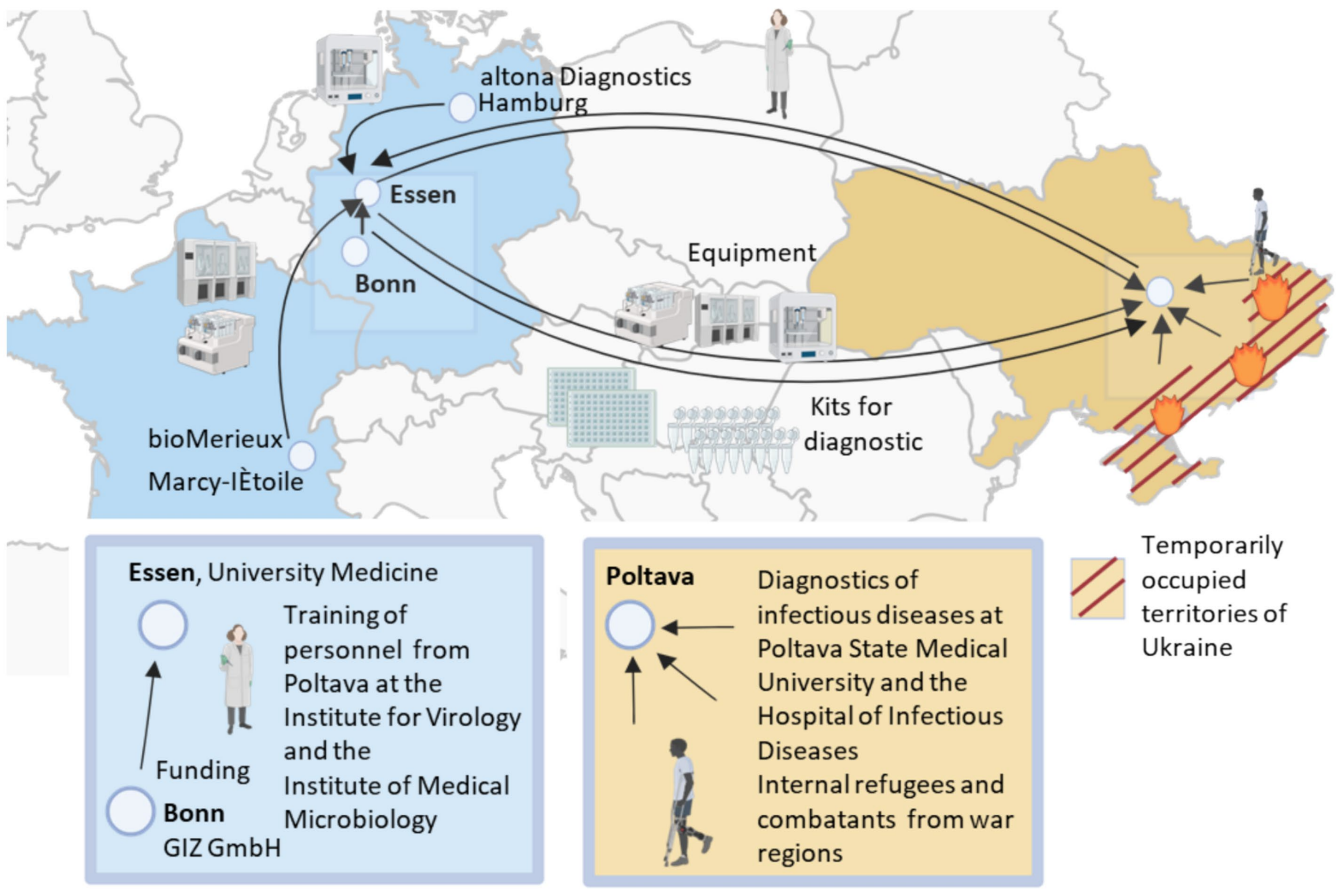
Cooperation between Germany and Ukraine to strengthen infectious disease diagnostics. The project supports the Poltava State Medical University and the Hospital of Infectious Diseases through the German partner institutions in Essen. The project includes providing diagnostic equipment and kits and training specialist staff in Essen. Industry partners who provided the equipment and kits were also involved in the project. The goal was the establishment and improvement of diagnostics in the Poltava for the most common and medically relevant infectious diseases among internal refugees and combatants from war zones in eastern Ukraine. All relevant assay systems were successfully established in the year 2024. The figure was created in BioRender.

Between June and December 2023, PSMU and Poltava Regional Clinical Infectious Diseases Hospital received the major diagnostic devices and equipment (Figure 1). The delivery of reagents and consumables started in January 2024. Subsequently, several CE-marked diagnostic tests for infectious diseases were established, validated, and implemented in Poltava, allowing their consistent application (Table 1). For the procurement and delivery of diagnostic equipment and consumables, the established logistical channels were employed. Collaboration with Ukrainian logistics companies enabled the direct supply of materials to PSMU and the Poltava Regional Clinical Infectious Diseases Hospital. Because of the ongoing attacks by the Russian army on the Ukrainian power supply system, including the Poltava region, generators were delivered from Germany to stabilize cold chains and work processes. Security aspects were carefully considered during both the planning and implementation phases of the project. The project strategy focused on ensuring physical continuity of laboratory services under wartime conditions, including the use of backup generators and temperature-controlled storage during power outages. To mitigate single-point failure risks, critical equipment was distributed across two separate locations in Poltava, ensuring operational redundancy if one site is compromised. To avoid the risk of cyberattacks, all laboratory equipment operates offline without external network connections.

**Table 1:** Summary of diagnostic tests performed in Poltava between March 2024 and June 2025.

*MINI VIDAS*			
Diagnostic kit name	Performed tests	Thereof positive D	Thereof positive (%)
VIDAS HIV DUO Ultra	3,915	63	1.6
VIDAS HBs Ag Ultra	2,585	124	4.80
VIDAS Anti-HCV Ultra	2,707	421	15.55
VIDAS HAV IgM m-capture	660	46	6.97
VIDAS Toxo IgM m-capture P30 Ag	240	1	0.42
VIDAS Toxo IgG	240	77 (thereof 2 doubtful)	34.40
VIDAS Toxo IgG Avidity	180	62	34.44
VIDAS QCV Test	814		
VIDAS Lyme IgM	240	28 (thereof 3 doubtful)	11.70
VIDAS Lyme IgG	240	17	7.08
VIDAS VCA IgM (conducted at PSMU)	92	0	0
VIDAS VCA IgG (conducted at PSMU)	92	88	95.60
VIDAS EBNA IgG (conducted at PSMU)	92	85	92.40
VIDAS CMV IgM (conducted at PSMU)	81	1	1.20
VIDAS CMV IgG (conducted at PSMU)	92	84	91.30

*VITEK 2*			
Diagnostic kit name	Performed tests	Thereof positive D	Thereof positive (%)
AST-Р 644 test	753	753	
VITEK 2 YST ID yeasts	278	278	
VITEK 2 GP ID gram positive	558	558	
VITEK 2 GN ID enterobacteria	800	800	
AST-N 429 test	566	566	
VITEK 2 NH identification	5	5	
VITEK 2 ANC identification	7	7	
AST-N 330 test	857	857	
AST-P 643 test	252	252	

*altoStar*			
Diagnostic kit name	Performed tests	Thereof positive D	Thereof positive (%)
HBV	35	6	17.14
HCV	40	6	15
Adenovirus	4	0	0
Norovirus	26	0	0
HSV-1/2	10	0	0
EBV	64	8	12.5
CMV	65	8	12.31
HHV-6	43	0	0

All test performed on the diagnostic systems *MINI VIDAS*, *VITEK 2*, and *altoStar* are shown. Overall, 16,633 test were performed in 15 month.

The selection of the MINI VIDAS (bioMérieux), VITEK 2 (bioMérieux), and altoStar (altona Diagnostics) platforms was guided not only by diagnostic performance, but also by operational suitability for the wartime healthcare context in Ukraine. Key decision criteria included robustness of the systems, compatibility with existing laboratory infrastructure, availability of CE-marked assays, ease of implementation and staff training, reliability under unstable power supply conditions, feasibility of maintenance and technical support, sustainable reagent supply through established Ukrainian distribution channels, and overall cost-effectiveness. In comparison with larger high-throughput systems, these platforms were considered more practical and economically sustainable for medium-capacity regional laboratories operating under constrained logistical and infrastructural conditions. Furthermore, the selected systems support standardized workflows compatible with internationally recognized laboratory quality principles and future alignment with ISO 15189-oriented accreditation pathways and relevant European *in vitro* diagnostic regulatory frameworks (39).

Compared with previously in Poltava utilized rapid diagnostic tests (RDTs) for the detection of HBV, HCV, and HIV, the diagnostic approaches implemented within this project demonstrate substantially improved performance. Although the World Health Organization (WHO) recommends the use of RDTs for clinical testing—particularly in resource-constrained settings and among vulnerable populations—these assays typically exhibit lower sensitivity and specificity (40) than advanced diagnostic platforms. Consequently, RDTs are more susceptible to false-negative results, leading to missed infections, as well as false-positive results, which can hinder the accuracy of diagnosis and subsequent patient management and treatment in public health practice (41).

The introduction of the *MINI VIDAS* test system represents a notable advancement in the screening and diagnosis of HBV, HCV, and HIV relative to the commonly used rapid tests in Ukraine. *MINI VIDAS* is an automated immunoassay platform utilizing Enzyme-Linked Fluorescent Assay (ELFA) technology, recognized for delivering markedly higher sensitivity and specificity than RDT-based methodologies. The Poltava Regional Clinical Infectious Diseases Hospital has started to operate the *MINI VIDAS* equipment (bioMérieux) for serological tests in March 2024. The *VITEK 2* equipment (bioMérieux) for microbial identification (ID) and antibiotic susceptibility testing (AST) started operating in June 2024. The *altoStar* (altona Diagnostics) for PCR tests started operating in October 2024. Before the altoStar based test preparation could be established a technical support person had to be trained at Altona diagnostics in Hamburg, Germany. On a monthly basis, test reports were shared and jointly discussed between the partners. Test results generated until June 2025 were included in this publication. Tests for Epstein Barr-Virus (EBV) and Cytomegalovirus (CMV) using the *MINI VIDAS* were mainly performed at the PSMU laboratory. Reports from Poltava provided data on the number and types of diagnostic tests performed. This regular communication allowed for continuous assessment of actual needs and consumption patterns. Accordingly, the purchasing strategy and subsequent supply of diagnostic reagents were adjusted to reflect the real-world situation in Poltava, ensuring uninterrupted diagnostic capacity and efficient resource utilization.

A notable rise in viral hepatitis cases was observed in the Poltava region after the project started. Testing in the Poltava Regional Clinical Infectious Diseases Hospital revealed a substantial number of patients with markers for HBV or HCV infections or even both infections. Many patients, including those returning from military service, showed signs of active or resolved viral infections. Overall, the findings point to a high burden of hepatitis virus infections in patients preselected for infectious disease diagnostics.

## Discussion and lessons learned

### Establishment of EU-compatible diagnostic infrastructure and capacity building

Efficiently diagnosing infections during wartime is crucial because armed conflict dramatically increases the risk of infectious diseases due to displacement, poor hygiene, and stress on the population (4). At the same time, warfare can weaken or destroy health systems by damaging infrastructure, disrupting supply chains, and reducing the availability of trained medical personnel (42). Wounded combatants and civilians are especially vulnerable because injuries, malnutrition, and limited access to care make them highly susceptible to infectious diseases (43). Within this context, our project successfully established an EU-compliant diagnostic infrastructure for infectious diseases under wartime conditions in Poltava, Ukraine, while simultaneously strengthening local laboratory capacities and supporting long-term sustainability.

A central lesson learned was the importance of adapting implementation strategies to local technical, institutional, and logistical realities. Major operational challenges included administrative procedures in Germany, disruptions of transport routes caused by the war, unstable electricity supply, and organizational barriers related to staff mobility and training. These obstacles were addressed through flexible project management, close coordination between German and Ukrainian partners, reliance on Ukrainian logistics companies, and continuous reassessment of local needs. The use of local logistics partnerships enabled direct and comparatively stable delivery of diagnostic equipment, reagents, and consumables despite wartime disruptions. In parallel, generators supplied from Germany ensured continuity of laboratory workflows and cold-chain stability during repeated attacks on the Ukrainian energy infrastructure.

Another important lesson learned was the value of combining international training with local capacity building. Ukrainian physicians and laboratory personnel received intensive hands-on training in Germany focused on diagnostics, quality management, and standard operating procedures (SOPs). Language and travel-related barriers were mitigated through hybrid communication strategies, bilingual coordination, and long-term institutional collaboration between partners. The training of a dedicated technical support specialist for altoStar PCR workflows at altona Diagnostics in Hamburg proved particularly important for ensuring sustainable local operation and troubleshooting capacity after implementation in Poltava.

By late 2023 and early 2024, diagnostic equipment was successfully delivered and implemented in Poltava, enabling advanced testing in EU-compliant diagnostic facilities. The introduction of the *MINI VIDAS* system significantly improved the accuracy and reliability of HBV, HCV, and HIV screening diagnostics compared to previously used rapid tests (40). The implementation of modern serological, PCR and bacteriological tests into the routine of the KP Poltava Regional Infectious Diseases Hospital has greatly expanded the diagnostic capabilities for several different, clinically relevant infections. The ELFA test for blood-born infections (combined antibody/antigen test) significantly improved the specificity and sensitivity of the diagnosis compared to rapid tests (40), which had been applied for routine practice before (44). Overall, the implications of the improved diagnostics on public health in the Ukraine are meaningful (4, 45).

### Impact on public health and medical education

Outside of our project, serological tests for Toxoplasmosis, Herpesvirus infections, as well as PCRs detecting HBV and HCV are usually restricted to private laboratories and are very expensive for patients in the Ukraine. The obligation to pay for diagnostic procedures reduces the willingness of patients to seek medical advice and treatment. Therefore, the implementation of these tests within the framework of this project has significantly increased the local capacities to diagnose infectious diseases in the Poltava region and significantly improved patient care. So far, laboratory tests were not conducted for children, given that the hospital provides care only for adults.

Our project may also improve patient treatment in Poltava. The time it takes to detect antibiotic resistance is crucial for treating patients. The application of the *VITEK 2* semi-automated bacteriological analyzer noticeably improved diagnostic speed, reducing pathogen identification and the antibiotic susceptibility testing time from 72 to 48 h. This advancement not only enabled faster detection of pathogens but also greatly improved the accuracy, sensitivity, and precision in identifying antibiotic resistance (45). However, the high levels of antibiotic resistance observed—especially among Acinetobacter spp. and *E. coli*—highlight a pressing public health concern in the Ukraine that demands stronger infection control and antibiotic stewardship efforts (46).

Apart from clinical routine diagnostics, the implementation of two sets of *MINI VIDAS*, *VITEK 2,* and *altoStar* provides the scientific and educational foundation for research and teaching at the Research Institute of Genetic and Immunological Bases of Pathology and Pharmacogenetics, PSMU, for undergraduate, postgraduate and PhD students. This was very helpful to educate the next generation of physicians in state-of-the-art infectious disease diagnostics and treatment. The modern equipment offers medical students in Poltava enhanced opportunities for the practical application of knowledge acquired through the Online Essen-Poltava Winter Academy course on Infection and Immunity.

From a broader perspective, our experience suggests that successful implementation of advanced infectious disease diagnostics in conflict settings depends heavily on strong local partnerships, flexible logistics, redundancy planning for electricity and cold-chain stability, practical technology selection, and continuous communication between all stakeholders. These lessons may be transferable to other conflict-affected or resource-limited regions seeking to establish resilient diagnostic infrastructure under crisis conditions.

## Conclusion

The prototypical deployment of new serological and molecular diagnostic tools through the German-Ukrainian partnership represents a successful model that could be replicated in other regions of Ukraine. In the Poltava region, the introduction of these enhanced diagnostic capabilities has significantly improved the detection, monitoring and treatment of infectious diseases though it is currently limited to the adult population. Even under the challenging conditions of the ongoing war, this advancement has enabled more precise and personalized patient care. In addition, by facilitating the early and accurate identification of pathogens, it plays a critical role in reducing the transmission of infectious diseases, thereby strengthening overall public health resilience. This project demonstrates not only immediate clinical value but also strategic long-term impact for Ukraine’s health system. By strengthening diagnostic capacity in line with EU standards, it supports the country’s trajectory toward EU accession, particularly in harmonizing laboratory practices, antimicrobial resistance monitoring, and quality assurance frameworks. Beyond its current scope, the model offers a foundation for expansion into pediatric populations and integration into a national surveillance system, enabling more comprehensive and coordinated public health responses.

Crucially, this initiative shows that resilient, cost-effective diagnostic solutions are achievable even in conflict-affected settings. Its scalability and adaptability make it a compelling model for broader application. We therefore urge international funders, policymakers, and governments to prioritize and invest in similar approaches, ensuring that vulnerable regions are not left behind in accessing essential diagnostic infrastructure. Sustained commitment will be key to transforming short-term interventions into long-lasting health system improvement. Future expansions of this diagnostic model must include pediatric populations and epidemiological studies to ensure comprehensive infectious disease control across the whole Ukraine.

## Data Availability

The original contributions presented in the study are included in the article/supplementary material, further inquiries can be directed to the corresponding authors.

## Glossary

AMR: Antimicrobial Resistance
ANC: Anaerobic and Corynebacterium identification card
AST: Aspartate Aminotransferase
COVID-19: Coronavirus Disease 2019
CRP: C-Reactive Protein
DNA: Deoxyribonucleic Acid
EBNA: Epstein–Barr Nuclear Antigen
EBV: Epstein–Barr Virus
EC: stands for Conformité Européenne (French for “European Conformity”)
ECDC: European Centre for Disease Prevention and Control
ELFA: Enzyme Linked Fluorescent Assay
EU: European Union
GIZ: Deutsche Gesellschaft für Internationale Zusammenarbeit
HAV: Hepatitis A virus
HBV: Hepatitis B Virus
HCV: Hepatitis C Virus
HEV: Hepatitis E Virus
HHV-6: Human Herpesvirus 6
HIV: Human immunodeficiency virus
HPV: Human Papillomavirus
IgG: Immunoglobulin G
IgM: Immunoglobulin M
MIC: Minimum Inhibitory Concentration
PLWH: People Living with HIV
PCR: Polymerase Chain Reaction
PSMU: Poltava State Medical University
SARS-CoV-2: Severe Acute Respiratory Syndrome Coronavirus 2
STI: Sexually Transmitted Infections
SOP: Standard Operation Procedures
TB: Tuberculosis
UME: University Medicine Essen
UNAIDS: Joint United Nations Programme on HIV/AIDS
US PEPFAR: United States President’s Emergency Plan for AIDS Relief
WHO: World Health Organization
WZI: West German Center for Infectious Diseases (German for “Westdeutsches Zentrum für Infektiologie”)
